# Integrated TB and HIV care for Mozambican children: temporal trends, site-level determinants of performance, and recommendations for improved TB preventive treatment

**DOI:** 10.1186/s12981-020-00325-9

**Published:** 2021-01-09

**Authors:** W. Chris Buck, Hanh Nguyen, Mariana Siapka, Lopa Basu, Jessica Greenberg Cowan, Maria Inês De Deus, Megan Gleason, Ferreira Ferreira, Carla Xavier, Benedita Jose, Criménia Muthemba, Beatriz Simione, Peter Kerndt

**Affiliations:** 1 David Geffen School of Medicine, University of California Los Angeles, Los Angeles, USA; 2 London School of Hygiene and Tropical Medicine, London, UK; 3 Impact Epilysis, Thessaloniki, Greece; 4 United States Agency for International Development, Maputo, Mozambique; 5 Centers for Disease Control and Prevention, Maputo, Mozambique; 6 National Tuberculosis Control Program, Ministry of Health, Maputo, Mozambique; 7 National HIV Program, Ministry of Health, Maputo, Mozambique

**Keywords:** Pediatric, Children, HIV, Tuberculosis, Co-infection, IPT, Cotrimoxazole, ART

## Abstract

**Background:**

Pediatric tuberculosis (TB), human immunodeficiency virus (HIV), and TB-HIV co-infection are health problems with evidence-based diagnostic and treatment algorithms that can reduce morbidity and mortality. Implementation and operational barriers affect adherence to guidelines in many resource-constrained settings, negatively affecting patient outcomes. This study aimed to assess performance in the pediatric HIV and TB care cascades in Mozambique.

**Methods:**

A retrospective analysis of routine PEPFAR site-level HIV and TB data from 2012 to 2016 was performed. Patients 0–14 years of age were included. Descriptive statistics were used to report trends in TB and HIV indicators. Linear regression was done to assess associations of site-level variables with performance in the pediatric TB and HIV care cascades using 2016 data.

**Results:**

Routine HIV testing and cotrimoxazole initiation for co-infected children in the TB program were nearly optimal at 99% and 96% in 2016, respectively. Antiretroviral therapy (ART) initiation was lower at 87%, but steadily improved from 2012 to 2016. From the HIV program, TB screening at the last consultation rose steadily over the study period, reaching 82% in 2016. The percentage of newly enrolled children who received either TB treatment or isoniazid preventive treatment (IPT) also steadily improved in all provinces, but in 2016 was only at 42% nationally. Larger volume sites were significantly more likely to complete the pediatric HIV and TB care cascades in 2016 (p value range 0.05 to < 0.001).

**Conclusions:**

Mozambique has made significant strides in improving the pediatric care cascades for children with TB and HIV, but there were missed opportunities for TB diagnosis and prevention, with IPT utilization being particularly problematic. Strengthened TB/HIV programming that continues to focus on pediatric ART scale-up while improving delivery of TB preventive therapy, either with IPT or newer rifapentine-based regimens for age-eligible children, is needed.

## Background

Recent epidemiologic studies estimate that 2.1 million children are living with human immunodeficiency virus (HIV) globally [1]. As of 2016, approximately 108,000 children were living with HIV (CLHIV) in Mozambique, with an estimated 75,953, or 70%, on antiretroviral treatment (ART) [2]. The World Health Organization (WHO) estimated pediatric tuberculosis (TB) incidence in 2016 was 1.0 million cases globally, with 22,000 new pediatric cases in Mozambique [3]. In 2017, the Mozambique Ministry of Health (MoH) registered 11,198 pediatric TB cases (children 0–14 years of age), which represents a 51% case detection rate based on WHO epidemiologic estimates of overall childhood TB burden. Additionally, the MoH reported a 40% HIV-infection rate among registered adult and pediatric TB patients [4].

From these data, it is evident children in Mozambique account for a significant proportion of the global pediatric HIV and TB burden, and there are sizable gaps between the reported numbers of children receiving care and the estimates of those in need. Given the high rates of HIV-TB co-infection, well-integrated care programs for these diseases are critical to ensure that new cases are detected and that children receive the full package of available preventive and therapeutic evidence-based interventions [5–7]. This begins with routine screening, and both WHO and Mozambique national guidelines recommend active case-finding through provider-initiated testing and counseling (PITC) and/or routine opt-out testing for HIV in all children with TB, as well as routine symptom-based screening for TB during all pediatric HIV consultations [8–12].

For pediatric TB patients newly diagnosed with HIV, the evidence-based cascade of care includes starting cotrimoxazole preventive therapy (CPT) and timely initiation of ART (usually after two weeks of TB treatment) with regimens compatible with rifampicin-based TB therapy. These time-sensitive interventions are part of both WHO and national guidelines [10, 11, 13].

For children living with HIV, once active TB has been ruled out through routine screening, the risk of developing future disease can be reduced with TB preventive therapy (TPT). Accordingly, WHO and Mozambique national guidelines recommend isoniazid (INH) preventive therapy (IPT) in CLHIV over 12 months of age without active TB and newly enrolled in ART independent of contact history, and more targeted use in infants under 12 months of age without active TB but with a known contact [9, 12].

In Mozambique and other high-burden settings, well-integrated clinical management of children with HIV/TB co-infection following these guidelines continues to be problematic. Studies of HIV and TB co-infected children found high loss-to-follow-up rates among patients in both the HIV and TB care settings [14, 15]. There is also evidence demonstrating lower quality of HIV care (including proper TB screening, treatment, and prevention) is associated with lower retention and higher mortality [16]. TPT scale-up and treatment completion continues to be a challenge in most countries with a significant pediatric HIV epidemic [17]. And successful linkage of patients between the TB and HIV sectors faces persistent operational barriers [18–20].

As such, the overall aim of this study was to evaluate Mozambique pediatric TB-HIV national program results, identify strengths and weaknesses concerning adherence to national TB and HIV guidelines, and propose approaches that could be implemented to improve national program performance and pediatric outcomes.

## Methods

### Setting and data source

The President’s Plan for Emergency AIDS Relief (PEPFAR) supports the Mozambique MoH’s HIV and TB programs. The Centers for Disease Control and Prevention (CDC) and the United States Agency for International Development (USAID) provide PEPFAR funding to clinical partners who report routine data from both the HIV and TB programs. The frequency and breadth of data collected by the PEPFAR partners are greater than that collected routinely by the MoH, which often does not include age disaggregations. This study used PEPFAR aggregate data that were collected from both electronic patient tracking systems (ePTS) and paper-based patient registries at health care facilities.

### Design

A retrospective analysis of site-level, aggregate patient TB/HIV indicators for patients 0–14 years of age was conducted with data from 2012 to 2016. Due to differences in clinical partner data collection approaches during the time period of the study which affected full reporting of indicators of interest, only data from CDC-supported sites using ePTS and USAID-supported sites using paper-based registries were included. Health centers in the TB sector without at least one child co-infected with HIV were excluded, and from the HIV sector, sites without at least one child on ART were excluded.

### Analysis

The TB sector indicators included in the analysis were percentage of children tested for HIV, percentage of co-infected children who initiated CPT, and percentage of co-infected children who initiated ART. Indicators from the HIV sector included the percentage of children who were screened for TB at their last clinical consultation, the number of children treated for TB, and the percentage of new enrollees who received IPT or TB treatment. Trends in these indicators over the study period were reported as frequencies. A more detailed analysis of the most recent 2016 data was done to evaluate the performance of sector-specific care cascades relative to site-level independent/explanatory variables. These variables include province, district type (rural vs. urban according to standard MoH definitions), pediatric ART patient volume (converted into a categorical variable based on quartiles), and whether a site was a new or established pediatric ART site (established sites had at least one pediatric ART patient enrolled as of 2014, new sites enrolled their first pediatric ART patient in 2015 or 2016). The distribution of patients and sites according to these independent variables was analyzed with descriptive statistics including aggregate numbers of patients and sites with associated frequencies and a range for the lowest and highest site-level results.

The percentage of co-infected children with TB who initiated ART, and the percentage of newly enrolled CLHIV who received either TB treatment or IPT were chosen as the primary dependent outcomes of the TB and HIV care cascades, respectively, for tests of association with the site-level independent variables of interest. Data on initiation of IPT and TB treatment were analyzed; however, data about completion of treatment was not available. Generalized linear models with the logit link function and the binomial family were used to estimate the impact of each independent variable since both of our primary outcomes were bounded (0–100%). Separate models were constructed for each of the independent variables (unadjusted models) and adjusted models with all independent variables in the same model, with regression coefficients with 95% confidence intervals and p values reported. All models were adjusted for clustering within provinces to obtain robust standard errors using the vce(cluster) option in Stata. All analyses were performed in Stata version 14.0.a.

### Ethical considerations

Use of routine, anonymized data for this study was approved by the Mozambican Ethics Committee and the Center for Global Health Science Office at the Centers for Disease Control and Prevention. Informed consent was not required.

## Results

A total of 312 TB sites reporting 2350 co-infected children, and 718 HIV sites reporting 22,898 children newly enrolled in care (patients who commenced ART care for the first time), were included in the study. The majority of patients in both the TB and HIV cascade analysis were from rural (54.9% and 68.6%, respectively), high volume (83.4% and 68.3%, respectively), and established sites (92.3% and 86.9%, respectively). The distribution of patients and sites is presented in Table 1.

**Table 1:** Patient and site distribution according to independent variables, 2012–2016

	TB program (co-infected children)		HIV program (newly enrolled children)	
	Patients (N, %)	Sites (N, %)	Patients (N, %)	Sites (N, %)
All Sites	2350 (100.0%)	312 (100.0%)	22,898 (100.0%)	718 (100.0%)
Province				
Cabo Delgado	100 (4.3%)	23 (7.4%)	1846 (8.1%)	83 (11.6%)
Cidade De Maputo	229 (9.7%)	20 (6.4%)	1346 (5.9%)	26 (3.6%)
Gaza	340 (14.5%)	24 (7.7%)	2145 (9.4%)	77 (10.7%)
Inhambane	131 (5.6%)	15 (4.8%)	900 (3.9%)	22 (3.1%)
Manica	144 (6.1%)	18 (5.8%)	2942 (12.8%)	67 (9.3%)
Maputo	186 (7.9%)	30 (9.6%)	2133 (9.3%)	68 (9.5%)
Nampula	210 (8.9%)	45 (14.4%)	1946 (8.5%)	43 (6.0%)
Niassa	129 (5.5%)	17 (5.4%)	508 (2.2%)	38 (5.3%)
Sofala	306 (13.0%)	20 (6.4%)	4317 (18.9%)	88 (12.3%)
Tete	179 (7.6%)	31 (9.9%)	1876 (8.2%)	79 (11.0%)
Zambezia	396 (16.9%)	69 (22.1%)	2939 (12.8%)	127 (17.7%)
Setting				
Urban	1061 (45.1%)	85 (27.2%)	7196 (31.4%)	112 (15.6%)
Rural	1289 (54.9%)	227 (72.8%)	15,702 (68.6%)	606 (84.4%)
Patient volume^a^				
Quartile 1 (lowest)	69 (2.9%)	16 (5.1%)	940 (4.1%)	165 (23.0%)
Quartile 2	70 (3.0%)	28 (9.0%)	2206 (9.6%)	158 (22.0%)
Quartile 3	252 (10.7%)	83 (26.6%)	4117 (18.0%)	178 (24.8%)
Quartile 4 (highest)	1959 (83.4%)	185 (59.3%)	15,635 (68.3%)	217 (30.2%)
Experience^b^				
Established	2169 (92.3%)	278 (89.1%)	19,897 (86.9%)	511 (71.2%)
New	181 (7.7%)	34 (10.9%)	3001 (13.1%)	207 (28.8%)

^a^Based on pediatric ART patients, 2016

^b^New sites enrolled first pediatric ART patient in 2015 or 2016

### TB program trends

Our descriptive analysis at a national level indicates that 99% of TB patients (all ages, pediatric disaggregation not available from the study time period) were tested for HIV in 2016, up from 90% in 2012. CPT for co-infected children improved from 92% in 2014, 95% in 2015, and 96% in 2016. Initiation of ART for newly identified HIV co-infected children also showed steady improvement, from 81% in 2014, 86% in 2015, and 87% in 2016.

### HIV program trends

**Fig. 1:**
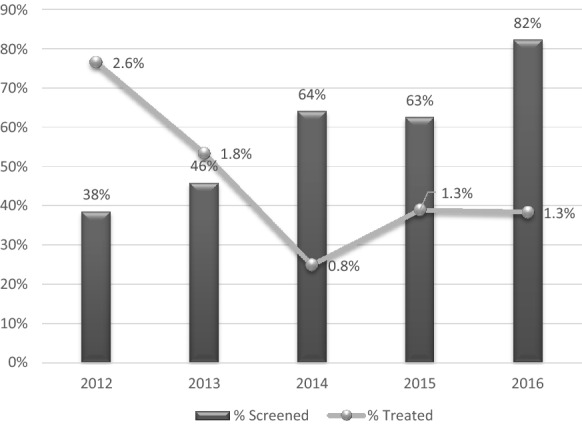
Percentage of CLHIV screened for TB at last clinical consultation and initiated on TB treatment

**Fig. 2:**
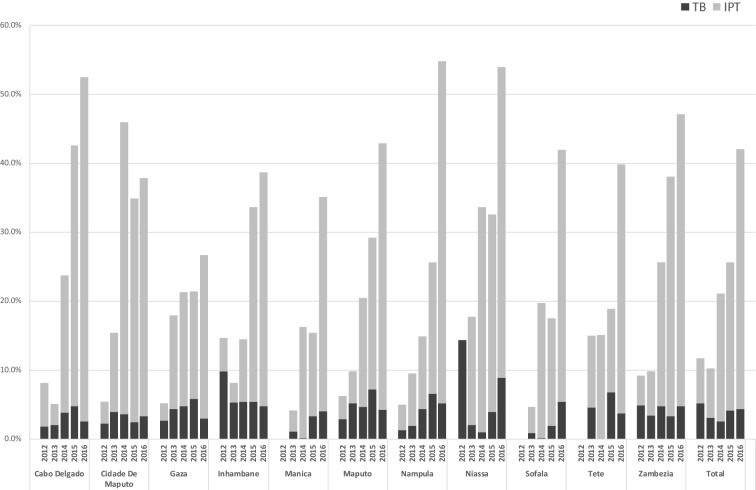
Newly enrolled CLHIV who received IPT or TB treatment by province, 2012–2016

Nationally, the percentage of HIV-infected children enrolled in care (includes newly enrolled and those already in care) and screened for TB at their last clinical consultation rose steadily from 2012 to 2016 (38% to 82%). The percentage of children diagnosed and treated for TB during the same time period did not rise correspondingly (2.6% to 1.3%), (Fig. 1). The number of newly enrolled HIV-infected children who received either IPT or TB treatment also trended positively in all provinces over the same time period, but remained well below target for 2016 at 42% nationally (37.7% received IPT and 4.3% received TB treatment) (Fig. 2).

### TB program sub-analysis

Using the percentage of co-infected children on ART as the end-point indicator for the TB sector care cascade, performance by province, care setting of urban and rural, patient volume, and site experience were analyzed. The results are detailed in Table 2. In unadjusted regression analysis, urban sites (p = 0.001) and those in the higher volume quartiles (p value range 0.043– < 0.001)) were significantly more likely to have co-infected children on ART, while in the adjusted analysis, only higher volume sites retained significance (p value range 0.015– < 0.001), with urban sites having near significant results (p = 0.05), (Table 3).

**Table 2:** Number and percentage of TB/HIV co-infected children ≤ 14 years on ART in the TB program in Mozambique, 2016

	# of co-infected patients on ART/Total # of co-infected patients (≤ 14 years)	(%)
All sites	2050/2350	87.2
Province		
Cabo Delgado	96/100	96.0
Cidade De Maputo	219/229	95.6
Gaza	284/340	83.5
Inhambane	123/131	93.9
Manica	134/144	93.1
Maputo	167/186	89.8
Nampula	189/210	90.0
Niassa	108/129	83.7
Sofala	254/306	83.0
Tete	138/179	77.1
Zambezia	338/396	85.4
Setting		
Urban	938/1061	88.4
Rural	1112/1289	86.3
Patient volume^a^		
Quartile 1 (lowest)	55/69	79.7
Quartile 2	55/70	78.6
Quartile 3	218/252	86.5
Quartile 4 (highest)	1722/1959	87.9
Experience^b^		
Established	1880/2169	86.7
New	170/181	93.9

^a^Based on pediatric ART patients, 2016

^b^New sites enrolled first pediatric ART patient in 2015 or 2016

**Table 3:** Linear regression for site factors associated with initiating ART in co-infected patients from the TB program, 2016*

	Univariable/Unadjusted Analysis		Multivariable/Adjusted Analysis	
	Reg Coeff (95% CI)	p value	Reg Coeff (95% CI)	p value
On ART				
Setting				
Rural	Reference	Reference	Reference	Reference
Urban	0.737 (0.293–1.182)	*0.001*	0.532 (− 0.001–1.065)	0.05
Patient volume^a^				
Quartile 1	Reference	Reference	Reference	Reference
Quartile 2	1.025 (0.033–2.017)	*0.043*	1.259 (0.246–2.272)	*0.015*
Quartile 3	1.053 (0.491–1.615)	< *0.001*	1.367 (0.736–1.999)	< *0.001*
Quartile 4	1.338 (0.581–2.095)	*0.001*	1.551 (0.762–2.40)	< *0.001*
Experience^b^				
New	Reference	Reference	Reference	Reference
Established	− 0.218 (− 1.295–0.860)	0.692	− 0.676 (− 1.664–0.312)	0.18

*Controlled for clustering of sites within provinces

^a^Based on pediatric ART patients, 2016

^b^New sites enrolled first pediatric ART patient in 2015 or 2016

### HIV program sub-analysis

Performance of percentage of newly enrolled HIV-infected children who received either IPT or TB treatment by province, setting, volume, and site experience are detailed in Table 4. In unadjusted regression analysis, volume quartile 2 was the only site-level variable significantly associated with IPT or TB treatment in newly enrolled children (p = 0.001), while in the adjusted analysis, sites in volume quartiles 1 and 3 had significant results (p =  < 0.001 and 0.043, respectively) (Table 5).

**Table 4:** Number and percent of newly enrolled children from the HIV program who initiated IPT or TB treatment in Mozambique, 2016

	IPT Initiation		TB Treatment	
	Number of Patients	2016 (%)	Number of Patients	2016 (%)
All Sites	8632/22,898	37.7%	992/22,898	4.3%
Province				
Cabo Delgado	923/1846	50.0%	46/1846	2.5%
Cidade De Maputo	465/1346	34.5%	44/1346	3.3%
Gaza	507/2145	23.6%	64/2145	3.0%
Inhambane	305/900	33.9%	43/900	4.8%
Manica	915/2942	31.1%	117/2942	4.0%
Maputo	823/2133	38.6%	91/2133	4.3%
Nampula	966/1946	49.6%	100/1946	5.1%
Niassa	229/508	45.1%	45/508	8.9%
Sofala	1577/4317	36.5%	233/4317	5.4%
Tete	678/1876	36.1%	69/1876	3.7%
Zambezia	1244/2939	42.3%	140/2939	4.8%
Setting				
Urban	2260/7196	31.4%	433/7196	6.0%
Rural	6372/15,702	40.6%	559/15,702	3.6%
Patient volume^a^				
Quartile 1 (lowest)	375/940	39.9%	24/940	2.6%
Quartile 2	927/2206	42.0%	39/2206	1.8%
Quartile 3	1816/4117	44.1%	106/4117	2.6%
Quartile 4 (highest)	5514/15,635	35.3%	823/15,635	5.3%
Experience				
Established	7359/19,897	37.0%	911/19,897	4.6%
New	1273/3001	42.4%	81/3001	2.7%

^a^Based on pediatric ART patients, 2016

^b^New sites enrolled first pediatric ART patient in 2015 or 2016

**Table 5:** Linear regression for site factors associated with IPT or TB treatment for newly enrolled patients from the HIV program, 2016*

	Univariable/unadjusted analysis		Multivariable/adjusted analysis	
	Reg Coeff (95% CI)	p value	Reg Coeff (95% CI)	p value
On either TB Treatment or IPT				
Setting				
Rural	Reference	Reference	Reference	Reference
Urban	− 0.018 (− 0.353–0.316)	0.914	− 0.013 (− 0.403–0.377)	0.947
Patient volume				
Quartile 1	Reference	Reference	Reference	Reference
Quartile 2	0.349 (0.143–0.554)	*0.001*	0.392 (0.209–0.575)	< *0.001*
Quartile 3	0.274 (− 0.087–0.635)	0.137	0.354 (0.001–0.706)	0.050
Quartile 4	0.124 (− 0.070–0.318)	0.210	0.226 (0.007–0.445)	*0.043*
Experience				
New	Reference	Reference	Reference	Reference
Established	− 0.068 (− 0.249–0.112)	0.459	− 0.165 (− 0.352–0.022)	0.084

*Controlled for clustering of sites within provinces

^a^Based on pediatric ART patients, 2016

^b^New sites enrolled first pediatric ART patient in 2015 or 2016

## Discussion

### TB program

This review of Mozambique national program data revealed that performance in the HIV care cascade from the TB program is strong, with positive trends and near-optimal performance in HIV testing (99.4%) and CPT initiation (95.7%) for co-infected children in 2016. ART initiation for co-infected children was lower (87.2%), but also improved from 2014 results of 81.0%. This indicator is limited in that co-infected children need to complete at least two weeks of TB treatment before initiating ART, so ART initiations in children enrolled near the end of a reporting period will be missed. That said, a result of 87.2% of pediatric TB patients newly identified as HIV + who started ART represents strong program performance and compares favorably with a 2015 global adult and pediatric rate of 78%, particularly since ART coverage rates for adults tend to be higher than for children [3].

In regression analysis controlled for provincial clustering, higher volume sites were significantly more likely to initiate co-infected children on ART. This is encouraging in that larger sites care for more of the overall number of affected children nationally, but, based on the results of this study, it is recommended that the MoH and supporting partners develop strategies to increase training and support to smaller sites to improve treatment outcomes.

### HIV program

Results from the HIV program are less encouraging and provide evidence of missed opportunities to reduce TB-related morbidity and mortality in HIV-infected children. Intensified case-finding, as measured by documented TB screen at the last HIV consultation, was 82.2% in 2016. This indicator is measured by a simple yes/no tick box on the standardized national pediatric ART patient record/mastercard (a clinical and monitoring tool for patients on ART) and it is impossible to assess whether these represent thorough TB screens.

One way to assess the quality and efficacy of TB screening is to monitor if the increased frequency of screening leads to increased diagnosis and treatment. In this review, the percentage of CLHIV in care who were diagnosed and treated for TB decreased from 2.6% in 2012 to 1.3% in 2016. It is possible that during the time of the study improved TB control activities, expansion of pediatric ART and IPT, and improved adult HIV and TB care could have resulted in decreased TB disease in CLHIV. As such, it is not possible to conclude that the reported TB screening in HIV-infected children was not high quality, but the findings do raise that possibility.

Another way to assess the quality of TB screening in this population is to consider the proportion of CLHIV newly enrolled on ART that was started on either IPT or TB treatment. The MoH does not have a formal goal for this indicator, but a reasonable benchmark would be 60% (55% IPT, 5% TB treatment) based on current trends in new enrollees and IPT age-based eligibility criteria. In this review, only 42.0% of newly enrolled children received either treatment in 2016, representing an over three-fold increase from 2012 results of 11.7%, and a positive trend despite the overall poor result. The major shortfall was with IPT, with only 37.7% of newly enrolled CLHIV receiving this preventive treatment.

Healthcare worker training that addresses known common misperceptions about the risk of INH toxicity, developing INH resistance while on IPT, and IPT efficacy and durability is recommended [21–28]. This training should also include content on how to address patients concerns about pill burden, adherence, and stigma and improving overall IPT treatment literacy [22, 29–31]. And Mozambique should consider fully aligning its TB screening algorithm with WHO recommendations that just use poor weight gain or loss, current cough, and fever to determine IPT eligibility [12].

Since the time period of this study, evidence has emerged that a three-month, once-weekly regimen of isoniazid and rifapentine (3HP) and a one-month regimen of daily isoniazid and rifapentine (1HP) have preventive efficacy similar to that of six months of isoniazid monotherapy while having superior outcomes in terms of adverse events and treatment completion [32, 33]. WHO has included 3HP and 1HP as alternative regimens for the treatment of latent TB in patients ≥ 2 years of age in recent guidelines (there is insufficient data to recommend rifapentine in children < 2 years at this time), and their adoption in Mozambique could undoubtedly help address some of the patient and healthcare worker barriers to IPT. However, the introduction of 3HP and 1HP will not resolve persistent challenges related to ruling out active TB in children, medical training gaps, and TB/HIV program integration.

### Limitations and strengths

This study has several limitations. The foremost is that the study used routine programmatic data that are intrinsically prone to error. However, PEPFAR performs routine data quality assessments and requires logic checks before partners can finalize and submit results. Despite the inclusion of pediatric HIV and TB indicators that are not available in the standard MoH reporting system, there were a limited number of site-level independent variables available for use in the regression analysis. Finally, there is an inherent bias towards larger established sites, as CDC clinical partners prioritized these health facilities for ePTS deployment. Larger sites did have better results in the TB care cascade and there may be an overestimation of aggregate national performance as a result. However, the sites included in the study accounted for the large majority of the children in care nationally, and thus the findings and recommendations should be more widely applicable.

## Conclusions

Pediatric TB, HIV, and TB-HIV co-infection are health problems with evidence-based diagnosis and treatment guidelines and well-defined cascades of care that should be followed in order to improve health outcomes among this already vulnerable population. The challenges facing children with these diseases in developing countries are less biomedical, in terms of defining what care should be delivered, but rather more operational, in terms of how to implement these recommendations in resource-constrained and overburdened healthcare systems.

This study demonstrates that Mozambique has made significant strides in improving the pediatric care cascades for children with TB and HIV. HIV case finding along with initiation of CPT and ART for co-infected children is systematically strong and trending positively. Results from the HIV sector, however, show significant missed opportunities for TB diagnosis and prevention, with IPT utilization being particularly problematic. While the site-level analysis did not reveal significant predictors of improved performance aside from higher patient volume, further province and district-level sub-analysis could help identify implementation best practices that could be adopted elsewhere.

Integrated TB/HIV programming that continues to focus on pediatric ART scale-up, while strengthening TPT can reduce morbidity and mortality in HIV-infected Mozambican children and opportunities for improved collaboration have been identified.

## Data Availability

The datasets used and/or analyzed during the current study are available from the corresponding author on reasonable request.

## Abbreviations

ART: Antiretroviral therapy
CDC: Centers for Disease Control and Prevention
CLHIV: Children living with HIV
CPT: Cotrimoxazole preventive therapy
ePTS: Electronic patient tracking system
HIV: Human immune deficiency virus
IPT: Isoniazid preventive therapy
MoH: Mozambique Ministry of Health
PEPFAR: President’s Emergency Plan for AIDS Relief
TB: Tuberculosis
TPT: Tuberculosis preventive therapy
TST: Tuberculin skin test
USAID: United States Agency for International Development
WHO: World Health Organization
